# The physical activity experience of prostate cancer patients: a multicentre peer motivation monitoring feasibility study. The Acti-Pair study

**DOI:** 10.1186/s40814-022-00966-9

**Published:** 2022-01-21

**Authors:** A. Baudot, N. Barth, C. Colas, M. Garros, A. Garcin, M. Oriol, F. Roche, F. Chauvin, N. Mottet, D. Hupin, on behalf the Acti-Pair investigators

**Affiliations:** 1 INSERM, U1059, SAINBIOSE, Université de Lyon, Université Jean-Monnet, Saint-Etienne, France; 2 Department of Clinical Investigation Centre, CIC 1408-INSERMClinical Investigation Centre, CIC 1408-INSERM, University Hospital of Saint-Etienne, Saint-Etienne, France; 3 Clinical Research Unit Innovation and Pharmacology, University Hospital of Saint-Etienne, Saint-Etienne, France; 4 Presage Institute - Hygee Centre, Université de Lyon, Université Jean Monnet, Saint-Etienne, France; 5 Chaire Santé des Ainés - Ingénierie de la prévention, Université de Lyon, Université Jean Monnet, Saint-Etienne, France; 6 Auvergne Rhône-Alpes (AURA) Gerontopole, University Hospital of Saint-Etienne, Saint-Etienne, France; 7 Department of Clinical and Exercise Physiology, University Hospital of Saint-Etienne, Saint-Etienne, France; 8 Sport-Health House, Departmental Olympic and Sports Committee of the Loire (42), Saint-Etienne, France; 9 National Centre for Health Examination Prevention (CETAF), Saint Etienne, France; 10 Department of Urology, University Hospital of Saint-Etienne, Saint-Etienne, France; 11 Department of Medicine Solna, Karolinska Institutet, Stockholm, Sweden

**Keywords:** Prostate cancer, Physical activity, Peer-mentoring, Accelerometry, Population Health Intervention Research

## Abstract

**Background:**

Although the benefits of physical activity (PA) on health are recognised, prostate cancer patients do not follow PA recommendations. The barriers to PA, whether physical, environmental or organisational, are known. Furthermore, even when such barriers are overcome, this achievement is not systematically accompanied by a change in lifestyle habits. The proposal of a programme enabling the integration of PA in the patient’s everyday life represents a new challenge in the personalized management of cancer patients. Peer-mentoring interventions have demonstrated their effectiveness in increasing adherence to PA by patients. This study aimed (1) to assess the feasibility of a peer-mentoring intervention: the Acti-Pair program in a local context and (2) to assess the effectiveness of the intervention in this context.

**Methods and analysis:**

A pre-post  design pilot study will be used to evaluate feasibility, potential effectiveness and implementation outcomes overs in prostate cancer patients. We performed a mixed quantitative and qualitative prospective study to assess means and process indicators and the implementation of the Acti-Pair program. This study will be performed in cancer centres of Loire district and will be comprised of three successive stages (1) diagnosis of the target population, (2) recruitment and training of peers, and (3) implementation of this intervention in the Loire department.

**Discussion:**

This study will allow us to extend the peer-mentoring intervention to other contexts and assess the effectiveness of this intervention and its generalisability.

**Supplementary Information:**

The online version contains supplementary material available at 10.1186/s40814-022-00966-9.

## Background

Prostate cancer is the most common male cancer in France (50,413 new cases per year in 2015) [1] and in Europe (365,000 new cases per year estimated in 2015) (https://ec.europa.eu/jrc/en/publication/epidemiology-prostate-cancer-europe). Recognised benefits of physical activity (PA) in tertiary prevention of prostate cancer include maintenance of autonomy, increased quality of life, and prolonged life expectancy [2, 3]. The beneficial role of PA regarding morbidity and mortality has already been demonstrated for patients with prostate cancer [4, 5]. Aerobic exercise may reduce prostate cancer progression and prostate cancer-specific death by influencing energy metabolism, inflammation, oxidative stress, and androgen receptor signalling pathways [6]. Accumulating evidence from prospective cohort studies suggests that PA, specifically moderate-to-vigorous physical activity (MVPA; i.e. activities that require an energy expenditure ≥ 3 times the resting metabolic rate, such as brisk walking or cycling), is associated with reduced risks of advanced, aggressive, and fatal prostate cancer [6]. Respect of new health standards (i.e. regular PA practice, diet, stress management, etc.) and lifestyle changes can be beneficial, even after 60 years of age [7]. Sedentary lifestyles, regardless of physical inactivity, increase the incidence of various cancers (breast, colorectal, endometrial and ovarian) and the risk of cancer-related mortality [8].

Given the strength of evidence, the promotion of PA and the fight against the sedentary lifestyle for people with chronic diseases is included in several national plans and programmes in France (National Nutrition and Health Programme 2011–2015 [9], National Health Strategy 2018–2022 [10], Cancer Plan 2014–2019) [11]. The World Health Organization (WHO) [12] recommends practicing 150 min of moderate intensity endurance activity (equivalent to brisk walking) per week. However, simply providing information about the benefits of this practice is not enough to change patients’ lifestyles.

Strengthening patients’ adherence to advice on PA in tertiary prevention is a challenge for personalised cancer care. The objective does not aim solely to treat cancer pathology but also to consider the patient as a whole, including all aspects of the patient’s life (including family, leisure and work activities), taking into account the person’s lifestyle in general with the objective of achieving a better quality of life. Although the benefits of PA on health are recognised, studies have shown that 60–70% of prostate cancer patients do not follow PA recommendations [13, 14]. It is recognised that prostate cancer patients are less adherent to PA practice recommendations than breast cancer patients [13, 14]. The identified barriers to the practice of regular PA by patients with prostate cancer are the following:

- Physical [15, 16] decline related to age or comorbidities, symptoms of the disease, undesirable side effects of the treatments, fatigue pain
- Psychological: personal efficacy, self-determination [17], former practice of PA, timing of the proposal given for PA following diagnosis [18], recommendations of PA by the oncologist [19], social support [20]
- Organisational: climatic conditions, temporal, geographical, or financial constraints [21].

These barriers partly explain the lack of PA in prostate cancer patients. However, overcoming these barriers is not systematically accompanied by a change towards a more active lifestyle. Encouraging people to participate in regular PA against the background of an inactive lifestyle is difficult, requiring attention to important psychosocial and behavioural influences [18, 22]. A major challenge is to provide a support structure for PA until this becomes a pattern of sustained healthy behaviour [23].

Peer-mentoring intervention helps survivors of pathological conditions to become physically active [24]. Peer mentors are individuals with similar salient characteristics, who have acquired experiential knowledge and are willing to share this [24]. Trinh et al. showed that a supervised 12-week PA programme continued to have motivational effects 5 years after the program had ended, including the perception of peer commitment [25]. Rabin et al. underlined the need for obtaining support from other patients who can understand their reactions to cancer [20]. Through the intervention of a peer, patients can rely on the support of a social environment and thus overcome the various psychological barriers identified in the literature (loss of self-esteem, lack of motivation, and competence) [26]. Peer-mentoring interventions have demonstrated their effectiveness in increasing adherence to MVPA by patients with diabetes [27], in older adults [28, 29], and more recently with breast cancer [30, 31] and prostate cancer patients [32]. They have also shown their effectiveness in promoting adherence to PA and long-term maintenance of PA by cancer patients [30, 31].

We hypothesised that an intervention combining three components helps to initiate and maintain in “real life” a behavioural change in prostate cancer patients with regard to the regular practice of PA:

1. Involving a peer mentor whose role is to provide social and motivational support by sharing his personal experience of the disease, its treatments, and the practice of PA with another patient.
2. Building a personalised and realistic PA project according to the patient’s needs, constraints, preferences, and environment. Evidence has shown that empowering patients to make their own choice as to which activity they intend to practice and where and with whom they will practice can improve PA level [33].
3. Benefitting from close support by healthcare professionals within the framework of a prescription for PA, as well as by PA professionals.

The available literature suggests that it is important to set up an intervention to promote and integrate PA in “real life” [34]. Rabin et al. specified the importance of flexibility in the implementation of PA programmes [20].

The primary objective of this pilot study will be to assess the feasibility, potential effectiveness and implementation of Acti-Pair programme for prostate cancer patients in the local context of the Loire department. Our primary objective and hypothesis will be related to feasibility outcome and will be to determine adherence to the Acti-Pair programme: the programme will be feasible if 60% of patients reach at least 60% of 1-h session of adapted physical activity (APA) as the minimum weekly threshold over the 3 months of intervention.

Secondary objectives will be to assess the potential effectiveness of the Acti-Pair program by measuring PA levels, quality of life, and implementation outcomes to inform a larger trial.

The purpose of this article is to describe the protocol of this study, following the SPIRIT reporting guidelines [35].

## Methods and analysis

### Study design

**Fig. 1:**
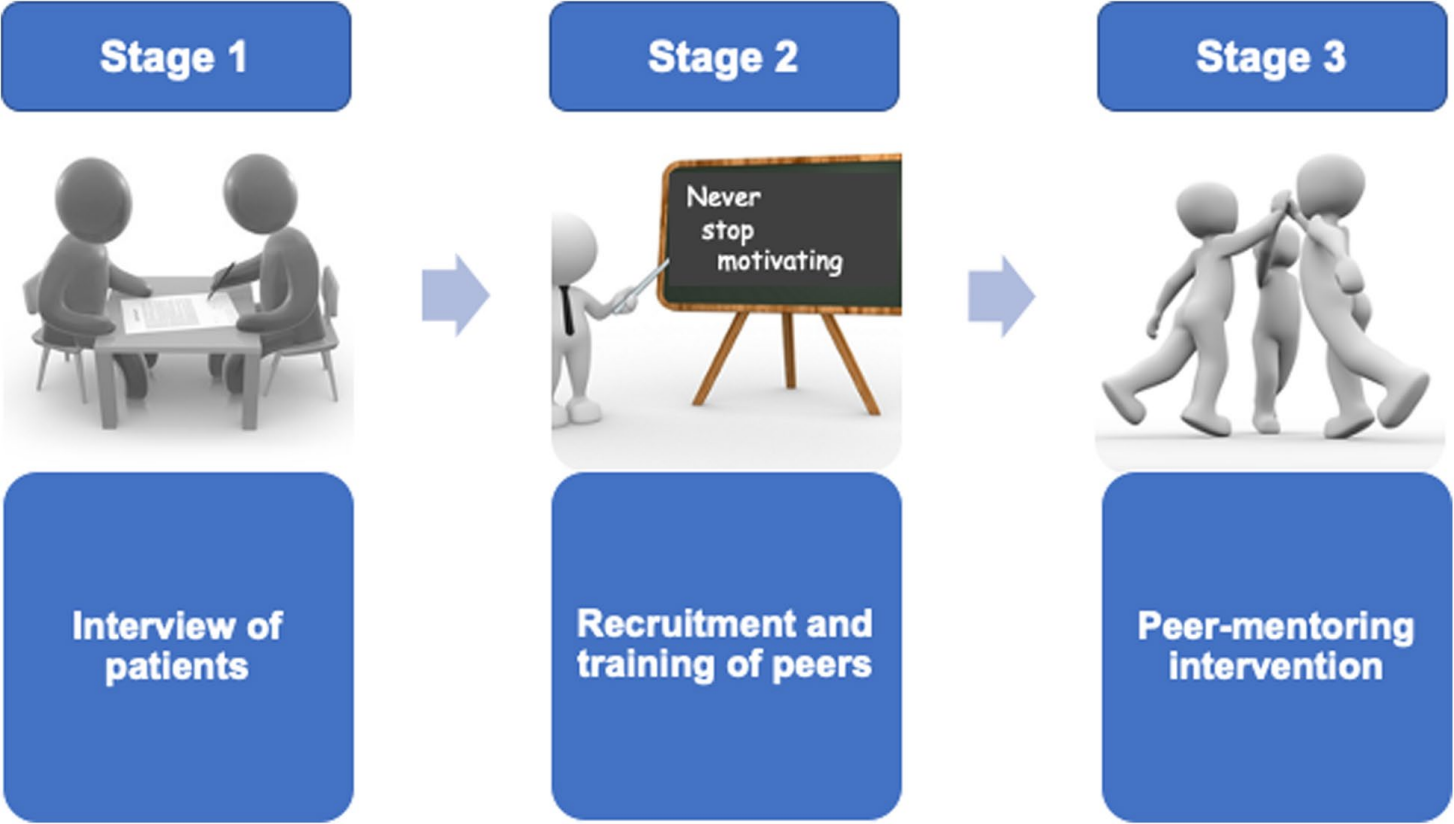
Study progress

This is a pre-post design pilot study using a mixed quantitative and qualitative methodology (*ClinicalTrials.gov**identifier: NCT03866785*). This French pilot study will be performed in cancer centres of Loire district and will be comprised of three successive stages: (1) diagnosis of the target population, (2) recruitment and training of peers, and (3) implementation of this intervention in the Loire department (Fig. 1).

For these stages, eligible adult patients will be consecutively included after their follow-up consultation with their oncologist or urologist.

1. In the first stage, the aim will be to identify the context of PA management and all the factors that facilitate the patient’s involvement in a more active lifestyle. It will be explored through one-on-one interviews.
2. In the second stage, peers will be trained to acquire the knowledge and skills to perform motivational follow-up of patients.
3. In the third stage, eligible patients will be mentored by peers. This stage will evaluate the reach and efficacy of the peer intervention by using before and after comparisons. This stage will be conducted with a sports and health platform of Loire department. This platform, labelled Sport-Health House in January 2020 by the French Ministry of Sports, is involved in a process of promoting clubs/associations offering a diversity of PAs adapted to (new) sedentary practitioners regardless of their age, level and form. This helps to improve the readability of health sport offers in the Loire department.

### Selection of participants

Patients and peers will be selected via two main hospitals in the Loire department treating patients with prostate cancer: the Lucien Neuwirth Cancerology Institute and the urology department of the St-Etienne University Hospital.

### Patients

The study will include consecutively adult (aged 18 years or older) patients with prostate cancer diagnosed at least one year ago and longer in the process of therapy. Individuals will be excluded from study participation if they are treated with hormone therapy and have cardiac or respiratory conditions contraindicating PA.

### Peers

Peers will be eligible and will have to meet the same inclusion criteria as patients, but they will also have to change their PA and sedentary behaviour permanently for at least 1 year. They will be physically active according to the WHO recommendations (150 min of MVPA per week).

The study will be conducted in accordance with the Declaration of Helsinki. Prior to starting the experiment, informed oral and written consents will be obtained from all participants. The protocol has been approved by the institutional review board (CPP Sud-Est I, France).

### Sample size

This is a feasibility study, so we will follow the general rule of thumb is to take 30 patients to estimate the (PA) observance for the target population [36]. We have chosen to include 10 peers and therefore 30 patients so that each peer follows a maximum of 3 patients. Intervention will concern 2 profiles according to the type of initial prostate cancer: (1) localised prostate cancer and locally advanced prostate cancer and (2) metastatic and/or resistant to castration and chemotherapy.

### The intervention: the Acti-Pair program

Intervention elements will be described using the Template for Intervention Description and Replication (TIDieR) checklist.

The Acti-Pair program proposes a combination of 3 intervention strategies, the aim of which is to initiate and maintain regular PA in prostate cancer patients:

1. Peer coaching (patient suffering from the same pathology and meeting the WHO recommendations for PA practice), which will provide motivational follow-up
2. The realisation of a personalized and realistic PA project via the PA platform systems
3. Support by health professionals (attending physician) via the prescription of PA.

### Diagnosis of the obstacles and levers to engage in a PA practice

Semi-structured interviews will be conducted by two sociologists specialising in APA, health, and ageing using common interview grids. They will be preferably conducted face-to-face and recorded using a dictaphone. They will be supported by a Master 2 trainee in public health for the processing of qualitative data (interviews, integral transcription, data analysis). Thirty interviews will be conducted until data saturation.

This comprehensive qualitative study will assess the following: (1) cancer disease and context/environment of subjects, (2) PA recommendations, representations and sports experience, (3) brakes and levers to resumption of PA following diagnosis of cancer; and (4) lifestyle changes since cancer diagnosis with multiple temporalities (future perspectives) and impact of the practice on the overall management of the disease (see supplementary data).

The goal of this diagnosis will be to have a better understanding of levers and obstacles of the target population about their PA practice.

### Recruitment and training of peers

The next phase will be the recruitment of peers. We have chosen to recruit them in the same centres as the target population in order to maximise the chances of having a similar geographical proximity and environment. The main role of peers will be to ensure motivational follow-up of patients in their PA project so that they initiate and/or maintain a regular and sustainable PA.

The peer patient's activities will be to (1) communicate with patients on a regular basis, (2) attend AP sessions, and (3) provide personalised assistance to each patient in order to help them to overcome obstacles in the realisation of the PA personalised project. Mentoring will be focussed on building a supportive relationship with patients, assessing motivational readiness, monitoring PA, identifying health concerns, and identifying and problem-solving barriers to PA, as realised in Pinto’s study [31].

Peer mentors will be trained to enable their acquisition of the knowledge and skills required to accomplish the motivational follow-up of patients. In particular, the objective of this training programme will be the acquisition of the following: (1) skills in PA counselling techniques (empathy, active listening) (imparted by a sports psychologist); (2) knowledge of functional signs or symptoms that might indicate a medical problem in order to ensure patient safety (imparted by a sports physician); and (3) knowledge required to determine heart rate and perceived effort rate in order to ensure an appropriate level of effort (imparted by an adapted physical activity professional, APA).

A qualitative interview will also be conducted to determine the obstacles and levers of the peer’s role before and after the intervention.

### 3-month intervention

Following baseline assessments (see Table 1), peer mentors will be matched to patients according to geographical proximity and similarity of cancer treatment. Peers will be instructed to call peers regularly for 3-months intervention and to conduct the first APA session physically with the patient. Peers can choose how they wish to support specific based on their preference, needs and constraints. Research staff will provide monthly individual supervision and guidance by phone during intervention delivery.

**Table 1:**
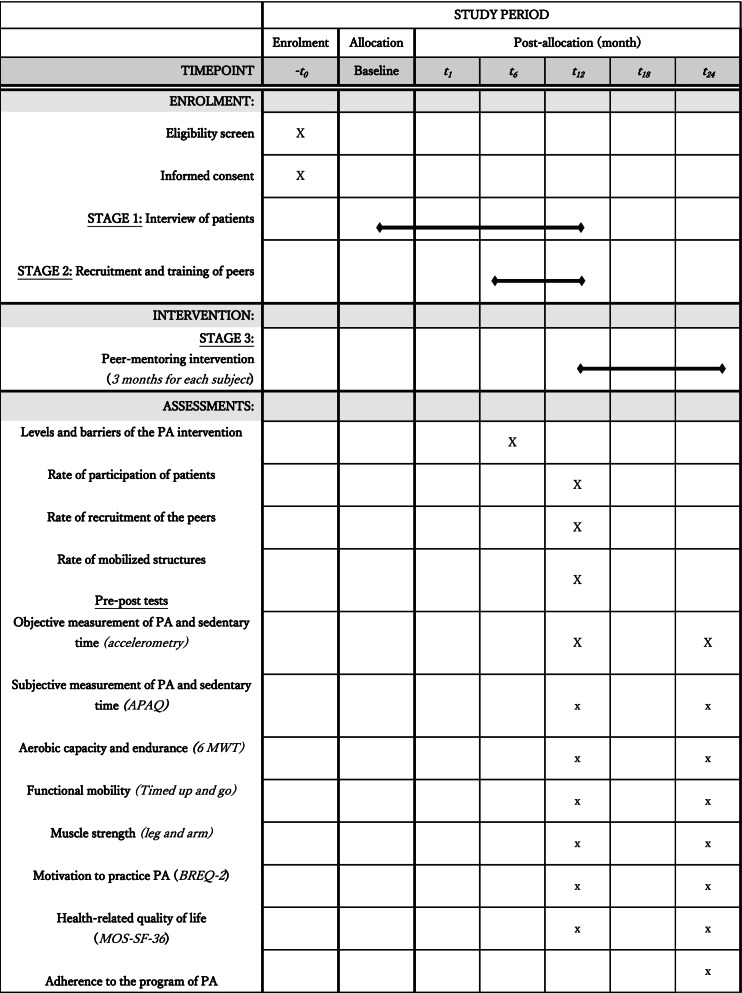
Summary and timing of assessments

*PA* physical activity, *APAQ* Adult Physical Activity Questionnaire, *BREQ-2* Behavioral Regulation in Exercise Questionnaire, *MOS-SF-36* Medical Outcome Study Form 36, *6 MWT* 6-min walk test

**Fig. 2:**
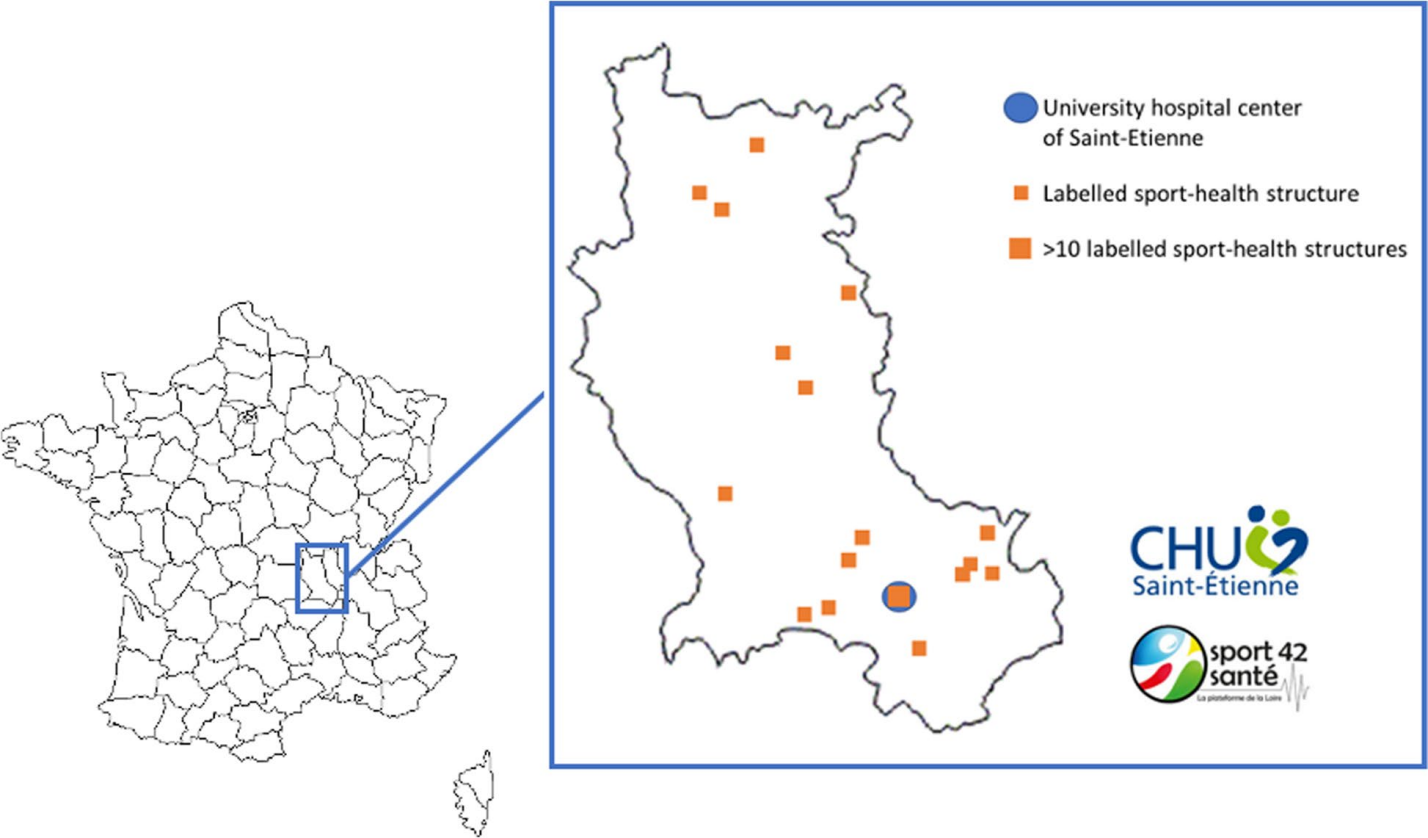
Labelled sport-health structures mapping

Peers will be supervised by the sports/health structure of their department throughout the intervention (Fig. 2). They will regularly update a field journal.

Prior to the intervention, each patient recruited will benefit from a personalised diagnosis by an APA professional within the sports/health structure: according to (i) the 6-min walk test (6 MWT) used to assess aerobic capacity and endurance, (ii) the timed up and go test which assesses functional mobility, (iii) the leg and arm strength tests, (iv) flexibility tests, and (v) postural balance. A patient will be able to choose the type and the place of the PA with whom he will practice cardiorespiratory endurance (cycling or brisk walking), muscle strengthening (gentle gymnastics, Pilates, gentle martial arts), or mixed activities (Nordic walking, martial arts, and sessions combining endurance and muscle exercises). The objective is to establish a personalised and realistic project, a key factor in ensuring the maintenance of long-term PA. A minimum of 1 hour of MVPA will be suggested. This PA project will gradually increase, and the further practice of PA will be planned with the APA professional. Patients will be asked to report symptoms, pain, and fatigue in a follow-up booklet, which will also contain tip sheets concerning PA. This booklet will facilitate shared follow-up of the patient by the various professionals involved and will thus constitute a communication tool.

Throughout the Acti-Pair intervention, meetings will be held with key stakeholders in order to promote sustainable investment for the provision of PA within the local community. These meetings will be scheduled every 6 months and will involve a discussion of needs, barriers to PA, and changes that could improve patient access and uptake of PA opportunities from the peer mentor’s point of view.

### Data collection

Each patient will wear an accelerometer (*ActiGraph GT3X*, Pensacola, Florida, USA) for 7 d before the start of the PA intervention to record his/her baseline level of PA and sedentary periods. Patients will also have to fill in an online questionnaire assessing PA and sedentary time (*Adult Physical Activity Questionnaire*, APAQ, EA SNA-EPIS, Jean Monnet University, Saint-Etienne, France) which will clearly distinguish the five components of PA (professional, travel, everyday life, leisure, sport) and will assess sedentary time. The motivation to practice PA will be assessed by the *Behavioural Regulation in Exercise Questionnaire-2 (BREQ-2)* [37] and *Medical Outcome Study Short Form 36 (MOS-SF-36)* used to assess health-related quality of life. At the end of the intervention, at 3 months, the patient will wear an accelerometer and will answer these same questionnaires.

The investigator-coordinator (DH) will have to declare to the quality management of the University Hospital Centre of St-Etienne, via the NORMEA software (http://intranet/Qualite/UrgencesRisques/normea/AccueilNormea.asp) any adverse events of trial conduct.

### Realistic evaluation

In this study, we will use a realistic evaluation approach based on Pawson and Tilley [38]. This theory driven approach focuses evaluation on the issue of what works, for whom and in what circumstances. Considering the evaluation design, multidisciplinary teams will be associated (public health teams, medicine sport science team, human and social sciences teams).

Both quantitative and qualitative data will be collected to perform the realist evaluation.

### Outcome’s assessments

#### Feasibility

- To assess the feasibility of the intervention, we will measure the number percentage adherence to the intervention will be measured by the number of patients pursuing PA 3 months after its initiation. We also assess the following: means indicators: rate of recruitment of the peers, rate of trained peers, rates of participation of patients in the intervention, rate of mobilised health professionals, rate of mobilised structures, number and type of involved professionals, training of the professionals in physical activity and the involved health professionals, peers and patients’ satisfaction.
- Process indicators: constraints obstructing the participation of peers and patients, constraints obstructing the participation of patients, recruitment method of peers and patients, type of PA, intensity, and duration.

#### Potential effectiveness

To assess the potential effectiveness of the intervention, we will measure objective measurement of PA (in MET-min/week; MET: metabolic equivalent, allows to measure the intensity of a PA), objective measurement of sedentary time (in h/d) via accelerometry, and subjective measurements of PA and sedentary periods using the *APAQ*. The value of this evaluation is to differentiate the five components of PA and to authenticate periods of inactivity and sedentary time, in addition to assessments using accelerometer; motivation to practice PA will be assessed by the *BREQ-2*, and health-related quality of life will be assessed by *MOS-SF-36*.

#### Implementation

The implementation will be analysis with a qualitative method which analyse conditions under which the intervention functioned. The analysis will focus on the following factors influencing implementation of the intervention and its progress:

- The characteristics of the peer mentors, determined by studying their profiles and the way they exercised their role with their assigned prostate cancer patients
- The interaction of the various stakeholders with the peer mentors in the context of their functions: the mode of support of prostate cancer patients and the moderating effects of the actors present in the intervention situations.

This assessment will be conducted by semi-directive interviews with peers, health professionals, APA professionals, representative of sports-health networks, and other stakeholders.

### Data analysis plan

#### Quantitative analysis

All collected individual variables will be described by frequency (%) for categorical variables and mean (SD), median (Q1-Q3), minimum, and maximum for quantitative variables. The data will be summarised separately in two tables: one for the period before intervention and another for the period after intervention.

The effectiveness of the intervention will be measured by frequency (%) of PA sessions conducted. Variations in MET-h/week for PA and h/week for physical inactivity will be assessed before and after patient intervention (peer coaching and regular PA practice). These same measurements will also be assessing peers before the training step and after the intervention.

These before/after variations will be compared in the same patient. These comparisons will be made using Student *t*-test for matched data if the distribution is normal. Otherwise, a rank test will be used.

#### Qualitative analysis

First, recorded interviews will be transcribed in verbatim. Transcripts will be read wholly and then line by-line to extract significant statements from the interviews, following established guidelines for a thematic analysis. These statements will be used to generate specific codes, and each transcript will then be coded using this thematic coding scheme. The themes emerging from the first interviews will help to refine the interview guide used for the next set of interviews. Data analysis will be performed simultaneously with *N-Vivo software* (*QSR international, Burlington, USA*) and continually with the data collection, in order to identify the arising of data saturation. The gathered information will then be categorised into five main themes based on the objectives of the study. Coding and extracted themes will be reviewed by a sport physician, a sociologist expert in qualitative research and a public health project manager.

Clinical Research Unit Innovation and Pharmacology (URCIP) from the University Hospital of Saint-Etienne (AB and AG) will be responsible of data monitoring and analysis.

The results of the study will be broadcast to participants, healthcare professionals and the public via information meetings and scientific publications (responsible: DH).

## Discussion

Acti-Pair combines several factors that have each independently demonstrated their effect on increasing PA in the long term. Acti-Pair aims (1) to assess the feasibility of a peer-mentoring intervention in a local context and (2) to assess the effectiveness of the intervention in this context. This study aims to integrate this intervention into the care offer for cancer patients by relying on existing city structures such as the sports health houses, labelled by the Ministry of Sports in January 2020, which make it possible to offer to patients a panel of PA according to their preference and also the geographical proximity to the structure (Fig. 2). Funding 3 months of intervention and adding motivational peer support would help perpetuate PA practice. The challenge is to go beyond hospital management and set up a “real life” PA to maximise the chances of PA sustainability. The proposed intervention would initiate and maintain regular PA to perpetuate it and thus increase the numerous benefits linked to the practice of regular PA: correction of physical deconditioning, metabolic and hormonal benefits, impact on quality of life, impact on the undesirable effects of treatments, and impact on the risk of recurrence and survival.

### Strengths and limitations

This pilot study will then allow us to extend the peer-mentoring intervention to other contexts and evaluate the effectiveness of this intervention and its generalisability. Acti-Pair will enable us to know the levels and barriers of the deployment of the intervention in various territories and thus to implement the intervention taking the various contexts into account. It will also allow the mobilisation of structures which propose different types of PA in different geographical locations around a common project. The objective of this intervention is to promote PA towards patients afflicted with cancer of the prostate. Such intervention could be developed thereafter vs. a control group for other types of cancers and chronic diseases. APA intervention will thus be integrated in the care pathway as a nonmedicinal therapeutic intervention complementary to other treatments. Its aim is to empower patients and thereby sustain the practice of PA. Including PA in the care pathway and in the everyday life of people suffering from chronic diseases and accompanying them towards an autonomous and sustainable practice of PA implies setting up cooperation between all actors within the health, social and medico-social, PA, and sports sectors.

Limitations of the pilot study include the small number of patients. The shorter lengths of recruitment and follow-up periods prevent us from examining the long-term maintenance of PA. In order to conclude on the effectiveness of the Acti-Pair program on the maintenance of regular PA for patients with prostate cancer, it will be necessary to set up a cluster stepped wedge randomised controlled trial.

## Supplementary Information

**Additional file 1.** Interview Guide for peers.

## Data Availability

Any data sharing requests should be sent to the corresponding author and would be subject to review and approval by the Clinical Research Unit Innovation and Pharmacology (URCIP) from the University Hospital Centre of Saint-Etienne and require data sharing agreements.

## Abbreviations

APA: Adapted physical activity
MVPA: Moderate-to-vigorous physical activity
PA: Physical activity
WHO: World Health Organization
