# Supplementary material for: The physical activity experience of prostate cancer patients: a multicentre peer motivation monitoring feasibility study. The Acti-Pair study
Source: Pilot Feasibility Stud. 2022 Jan 21;8:12. doi: 10.1186/s40814-022-00966-9 (PMC8781045; doi:10.1186/s40814-022-00966-9)
Supplement: Supplementary file 1 — Additional file 1. Interview Guide for peers. [file 40814_2022_966_MOESM1_ESM.docx]

**Interview Guide for peers – ACTI-PAIR**

| **THEMES** | **SUBTHEMES** | **QUESTIONS** | **REMINDERS** |
| --- | --- | --- | --- |
| **1. Cancer disease, management, representations** | Cancer diagnosis | I would like you to tell me your story with your cancer.  Can you tell me about how you found out you had cancer?  What was your reaction at the time of diagnosis?  Do you have any other health problems? | -Did you have any warning symptoms?  -Since when were you diagnosed?  -Who announced the pathology to you and under what circumstances?  -In the case of multiple pathologies (cancer, heart problem, obesity, etc.), what do you think is most important?  -Are there elements in your personal life that can explain the appearance of these pathologies? |
|  | Representation of prostate cancer | What did cancer mean to you (at the time of diagnosis)? | -Did you know about the disease?  -Do you know people who have been diagnosed with prostate cancer before? |
|  | Influence on individual | Did this diagnosis influence your professional or family life?  What was the role of your family in this ordeal? | -What changed at the time of diagnosis?  -How did they react? Were they supportive of you? |
|  | Medical/hospital management of disease | Can you tell me about the start of your medical care?  Evolution of the disease, treatment, recurrences? What are the medical recommendations? | -Who took charge of your pathology?  -How did that happen ?  -What is your compliance with these recommendations?  -Orientation towards care and education systems? |
| **2. Physical activity recommendations,**  **representations and sports experience** | APA recommendations | I would like to come back to these APA recommendations more specifically. Have you been recommended to practice regular PA?  How did you react to these recommendations? | -Who told you to do PA? Your GP, your oncologist or urologist ? Nurses ?  -Many times ? Never ?  -What do you think of these practice recommendations?  -Order of priority in medical recommendations (treatment, regular consultations, dietetic, etc.)? |
|  | Sports experience and representation of PA / sport / APA / physical practice at the time of diagnosis | Can you tell me about your experience in the field of PA before your cancer diagnosis?  What did "sport" mean to you? | -Were you athletic before the diagnosis? What kind of sport?  -Did you practice one or more sports when you were younger?  -Breakdown and continuity of PA / sport? Why?  -Are your loved ones athletic?  -What are your sporting goals (competition, performance, health) ? Evolution ? |
|  | Current state of practice, assessment of sedentary lifestyle | Do you practice regular PA today?  Do you do PA (gardening / DIY / housework, etc.)?  Do you consider yourself sedentary?  Are you a member of a patients association / sports association? | -If yes, which one?  -How frequently ?  -How many hours seated / day?  -Are you still in a professional activity? (Which job)  -If not, break in daily activities (energy expenditure) in retirement? |
| **3. Brakes and levers to resumption of APA following diagnosis of cancer** | Obstacles to restart an APA | Did you lessen barriers on resuming PA? (Constraints + difficulties encountered)  How did you overcome these initial difficulties? | Health reason? (Sickness, cumulation, pain, weakness, fatigue)  Lack of physical capacity? Feeling of incompetence?  Not suitable practice? Stressful practice?  Unsafe practice?  Expensive (economical) practice ?  Logistical problem ?  Family or work constraints (lack of time) ?  Fear of the judgment by others?  Other brakes? |
|  | Levers to restart an APA | What are the benefits of PA for you?  What are you looking for in your PA practice today? (Purpose, objectives) | Health? pleasure?  Secure, suitable environment?  Practice "between oneself", reassuring, not stigmatising?  Rewarding practice, taking control of your fragile body?  Social network (social benefits)?  Gain in strength, flexibility, vitality? (Physical benefits) |
|  | Stay engaged in an APA | What enabled you to maintain your commitment and even strengthen it?  What motivated you? | Group environment, professional role of APA? Capability?  Associative membership?  Do you think you have enough PA?  Would you like to do more?  Would you like to engage in another form of practice? |
|  | Resumption of PA (temporality, rupture and continuity) | How did you resume your PA following your cancer?  If you are a former sportsman, did you stop your practice following the diagnosis (or continue)?  Who are the actors who particularly helped you to invest in a PA?  How did the first sessions go? | In which structures / organisations? Patient education workshop devices? Sports association ? Autonomy ?  -What did these sessions consist of? Information / explanation / practice? What kind of PA?  Role of family, loved ones?  Role of health professionals?  Role of patients associations, sports patient associations?  Other influences (public health, media, etc.)  -Are you a volunteer?  -What did you like? Were you displeased? (Between you, APA teacher?) |
|  | Evolution of the practice following diagnosis and representations | How did your engagement in the practice of PA evolve?  Did you ever stop your practice?  If you have a sports history, how would you define your current practice compared to that before your diagnosis? | Rupture / continuity of the practice until today? Other PA? Other devices? Volunteering ?  How long have you been a member of an association?  How often (number of times per week)?  If yes, for what reason?  Do you sometimes encounter obstacles to practice? (Pain, fatigue, imperatives, etc.)  Did your concept of practice change? |
| **4. Lifestyle changes since cancer diagnosis** | Lifestyle development (before and after cancer) | Is there a before and after prostate cancer? | What did it change since the diagnosis?  -Medical care - Regular consultation?  -Everyday life ?  -Biographical disruption? (Virility)  -Willingness to take charge? |
|  | Evolution towards a more active lifestyle? | Is there a before and after practice of APA?  What place do you give to PA today? | -Do you see the impact of PA on other health components?  -New health standards / new lifestyle habits? (Food / adherence to treatment? / Relationship to the body?)  -Gain in autonomy?  -Is it an essential element in the management of your cancer? |
|  | Future prospects / feelings / morale / emotions | Have you changed your future prospects?  Do you think today that you are able to support people in a PA? | -What are your goals ? Your desires ? Your feelings?  -How would you define your morale today?  -Experience in sharing ? Moral support ? |

APA : adapted physical activity; PA: physical activity; DIY : do it yourself
